# Amine-reactive crosslinking enhances type 0 collagen hydrogel properties for regenerative medicine

**DOI:** 10.3389/fbioe.2024.1391728

**Published:** 2024-07-26

**Authors:** Daniel Salthouse, Peter D. Goulding, Sophie L. Reay, Emma L. Jackson, Chenlong Xu, Rochelle Ahmed, Andrew Mearns-Spragg, Katarina Novakovic, Catharien M. U. Hilkens, Ana Marina Ferreira

**Affiliations:** ^1^ School of Engineering, Newcastle University, Newcastle Upon Tyne, United Kingdom; ^2^ Translational and Clinical Research Institute, Newcastle University, Newcastle upon Tyne, United Kingdom; ^3^ Jellagen Limited, Cardiff, United Kingdom

**Keywords:** jellyfish-sourced type 0 collagen, hydrogels, chemical crosslinking agents, tissue engineering, regenerative medicine, mesenchymal stem/stromal cells

## Abstract

**Introduction:**

Collagen is extensively utilised in regenerative medicine due to its highly desirable properties. However, collagen is typically derived from mammalian sources, which poses several limitations, including high cost, potential risk of immunogenicity and transmission of infectious diseases, and ethical and religious constraints. Jellyfish-sourced type 0 collagen represents a safer and more environmentally sustainable alternative collagen source.

**Methods:**

Thus, we investigated the potential of jellyfish collagen-based hydrogels, obtained from Rhizostoma pulmo (R. pulmo) jellyfish, to be utilised in regenerative medicine. A variety of R. pulmo collagen hydrogels (RpCol hydrogels) were formed by adding a range of chemical crosslinking agents and their physicochemical and biological properties were characterised to assess their suitability for regenerative medicine applications.

**Results and Discussion:**

The characteristic chemical composition of RpCol was confirmed by Fourier-transform infrared spectroscopy (FTIR), and the degradation kinetics, morphological, and rheological properties of RpCol hydrogels were shown to be adaptable through the addition of specific chemical crosslinking agents. The endotoxin levels of RpCol were below the Food and Drug Administration (FDA) limit for medical devices, thus allowing the potential use of RpCol *in vivo*. 8-arm polyethylene glycol succinimidyl carboxyl methyl ester (PEG-SCM)-crosslinked RpCol hydrogels preserved the viability and induced a significant increase in the metabolic activity of immortalised human mesenchymal stem/stromal cells (TERT-hMSCs), therefore demonstrating their potential to be utilised in a wide range of regenerative medicine applications.

## 1 Introduction

Collagen is the major constituent of the extracellular matrix (ECM), providing structural support and flexibility. Indeed, collagen is one of the most widely utilised biomaterials in tissue engineering and regenerative medicine (Widdowson et al., 2018; Flaig et al., 2020; Ahmed et al., 2021; Liu, 2021; Riacci et al., 2021; Alves et al., 2022; Salthouse et al., 2023). Type I collagen is the most extensively used collagen and is commonly extracted from mammalian sources (typically of bovine or porcine origin) (Widdowson et al., 2018; Flaig et al., 2020; Ahmed et al., 2021; Riacci et al., 2021; Alves et al., 2022). However, mammalian-derived collagen poses several limitations, including high cost, risk of immunogenicity (associated to allergies), and potential transmission of viral vectors and infectious diseases such as bovine spongiform encephalopathy (BSE) and other transmissible spongiform encephalopathies (TSEs) (Widdowson et al., 2018; Flaig et al., 2020; Ahmed et al., 2021; Riacci et al., 2021; Alves et al., 2022). Furthermore, some patients refuse to receive treatment from mammalian-derived products due to ethical and religious reasons (Riacci et al., 2021; Alves et al., 2022). Therefore, marine-derived collagen is emerging as a promising alternative to mammalian collagen sources, as there is no risk of disease and viral vector transmission to humans, and is also a more environmentally sustainable collagen source than mammalian collagen (Widdowson et al., 2018; Flaig et al., 2020; Ahmed et al., 2021; Liu, 2021; Riacci et al., 2021; Alves et al., 2022). Jellyfish represent an attractive candidate as a non-mammalian source of collagen as they possess a high collagen content (more than 60% of their dry weight), and the global rise in jellyfish populations provides the potential to manufacture jellyfish-sourced type 0 collagen at a large scale (Widdowson et al., 2018; Flaig et al., 2020; Ahmed et al., 2021; Riacci et al., 2021). Jellyfish collagen is referred to as type 0 collagen due to its homogeneity to mammalian collagens, however, jellyfish collagen predates mammalian collagen by around 600 million years and is therefore biochemically simpler, allowing greater tissue functionality and structural versatility (Ahmed et al., 2021; Faruqui et al., 2023). Collagen from different species of jellyfish display similarities to different types of collagens (Song et al., 2006; Widdowson et al., 2018). For example, collagen derived from *Rhizostoma pulmo* (*R. pulmo*), commonly known as the barrel jellyfish, shows a high degree of similarity with collagen type I, while also demonstrating type II- “like” properties (Riacci et al., 2021). It is important to evaluate jellyfish collagen on a species level as different species of jellyfish possess differing factors that can impact the characteristics of collagen, including possessing different thermal stabilities due to experiencing different environmental temperatures for example (Widdowson et al., 2018). Different methods of collagen purification from different Mediterranean jellyfish species have been explored and it was concluded that *R. pulmo* provided the best yield of collagen and that this collagen displayed comparable biocompatibility to mammalian collagen (Addad et al., 2011; Flaig et al., 2020). *R. pulmo* collagen scaffolds have also demonstrated potential immunomodulatory and regenerative capabilities *in vivo* (Liu, 2021; Alves et al., 2022; Bowen et al., 2022; Faruqui et al., 2023).

Collagen-based hydrogels mimic the native ECM of tissues and can be injected into the body minimally invasively (in contrast to scaffolds), while chemical crosslinking agents can be incorporated to improve their mechanical stability and modulate degradation kinetics (Flaig et al., 2020; Ahmed et al., 2021; Riacci et al., 2021). Apart from few studies that have engineered *R. pulmo* collagen-composite hydrogels (Rigogliuso et al., 2020; Carvalho et al., 2022), there is limited knowledge on the chemical crosslinking and manufacturing of *R. pulmo* collagen-based hydrogels. To the best of our knowledge, *R. pulmo* collagen (RpCol) hydrogels have been only explored by incorporating different concentrations of genipin (Riacci et al., 2021). The authors found that higher genipin concentrations (2.5 mM and 5 mM) improved hydrogel stability, while lower genipin concentrations (1 mM) increased the metabolic activity of encapsulated human chondrocytes. Given the lack of reproducibility and manipulability issues of genipin-crosslinked RpCol hydrogels (Riacci et al., 2021), we performed a comprehensive exploration of alternative chemical crosslinking agents to understand and overcome these limitations. Therefore, this study produced a range of RpCol hydrogels by deploying a variety of chemical crosslinking agents and evaluated their potential to be utilised in regenerative medicine applications. Addressing a knowledge gap, we assessed the endotoxin levels of RpCol, which is essential due to potential pro-inflammatory responses *in vivo*. Additionally, we examined the effects of encapsulating immortalised human mesenchymal stem/stromal cells (TERT-hMSCs) within RpCol hydrogels on cellular behaviour and function. Our findings provide insights into the suitability of RpCol hydrogels for regenerative medicine, considering various physicochemical and biological properties.

## 2 Results and discussion

### 2.1 Physicochemical properties of RpCol hydrogels

**FIGURE 1:**
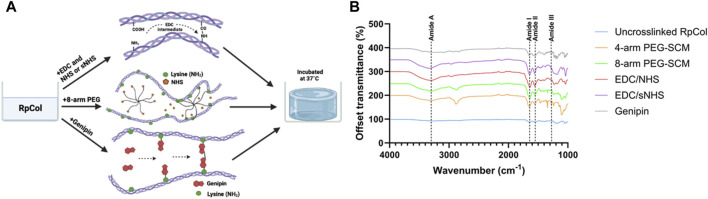
**(A)** Schematic representation of crosslinking of RpCol with selected chemical crosslinking agents; EDC with or without NHS or sNHS, multi-arm functionalised PEG-derivatives (8-arm shown), and genipin. Adapted from (Hwang et al., 2011; Rýglová et al., 2017; Fernandes-Cunha et al., 2020). Created in BioRender.com. **(B)** FTIR-ATR spectra of RpCol before (uncrosslinked RpCol) and after crosslinking with chemical crosslinking agents (4-arm PEG-SCM, 8-arm PEG-SCM, EDC/NHS, EDC/sNHS, and genipin) to form hydrogels.

RpCol hydrogels formed with several chemical crosslinking agents in various compositions were analysed to establish their potential for use in regenerative medicine applications. The schematic representation of the three crosslinking approaches that we deployed is depicted in Figure 1A: 1) carbodiimides (EDC (*N*-(3-Dimethylaminopropyl)-N′-ethylcarbodiimide hydrochloride) with or without NHS (N-hydroxysuccinimide) or sNHS (*N*-Hydroxysulfosuccinimide sodium salt)), 2) multi-arm functionalised polyethylene glycol (PEG)-derivatives, and 3) genipin.

To the best of our knowledge, the only study in the literature conducted on RpCol hydrogels used genipin as a chemical crosslinking agent (Riacci et al., 2021). Genipin can spontaneously react with primary amine groups of amino acid residues to form covalent bonds, leading to the formation of crosslinks (Hwang et al., 2011; Riacci et al., 2021; Reay et al., 2022), and has been shown to form stable RpCol hydrogels at specific concentrations (Riacci et al., 2021). However, due to the aforementioned issues described in that study, we also assessed several RpCol hydrogels that were synthesised using various different crosslinking agents. Both EDC and functionalised PEG-derivatives are widely utilised chemical crosslinking agents, both reported to be non-cytotoxic (EDC at low concentrations), and are typically able to produce stable structures (Rýglová et al., 2017; Widdowson et al., 2018; Fernandes-Cunha et al., 2020; Flaig et al., 2020; Ahmed et al., 2021). Therefore, we used a range of different compositions of EDC and PEG-derived crosslinking agents to form RpCol hydrogels and evaluated their resultant properties. There are numerous PEG-derived crosslinking agents with varying structures, number of arms, and molecular weights (MW). The structures of all PEG’s that we evaluated (5- and 20 kDa 4-arm PEG-GAS (polyethylene glycol succinimidyl glutaramide ester) and 10 kDa 4- and 8-arm PEG-SCM (polyethylene glycol succinimidyl carboxyl methyl ester)) all terminate with an NHS ester molecule, providing the crosslinking active site. These functionalised PEG molecules crosslink by binding to the -NH_2_ groups found on lysine residues, releasing the NHS molecule (Fernandes-Cunha et al., 2020). RpCol typically has more lysine residues than mammalian collagen (bovine and rat tail collagen type I) (Derkus et al., 2016; Carvalho et al., 2018; Carvalho et al., 2020) and thus has more potential crosslinking sites. The disadvantage of PEG-derivatives is that they can rapidly hydrolyse (when in contact with water) and lose their ability to crosslink (Shibayama et al., 2019). EDC crosslinks collagen by reacting the -COOH groups found on glutamic acid or aspartic acid residues with the -NH_2_ group found on lysine residues. This is facilitated by the formation of an intermediate product that can subsequently be hydrolysed into urea, which can be excreted from the body. NHS is commonly utilised in conjunction with EDC to stabilise the intermediate product and increase the extent of crosslinking, with varying concentrations and ratios of EDC/NHS typically deployed (Widdowson et al., 2018; Flaig et al., 2020; Ahmed et al., 2021). We therefore investigated a range of compositions of EDC/NHS to form a variety of RpCol hydrogels. The use of sNHS instead of NHS was also evaluated aiming to further increase the crosslinking efficiency, as this preserves or increases the water-solubility of the modified carboxylate molecule by virtue of the charged sulfonate groups on sNHS (Staros et al., 1986).

Fourier-transform infrared spectroscopy with attenuated total reflection (FTIR-ATR) can provide insights into the structure of collagen (before and after crosslinking) by generating spectra with distinct peaks (Paradiso et al., 2019; Reay et al., 2022; Reay et al., 2023). Amide A, I, II, and III peaks correspond to peptide bond vibrations, which are crucial to the formation of the triple helix, the fundamental characteristic structure of mammalian collagen responsible for its highly desirable properties (Belbachir et al., 2009; de Campos Vidal and Mello, 2011; Riaz et al., 2018; Felix et al., 2019; Paradiso et al., 2019). Figure 1B displays the representative spectra generated from RpCol before and after the addition of chemical crosslinking agents (16% (v/v) 4- and 8-arm PEG-SCM, 10% (v/v) EDC/NHS and EDC/sNHS 1:1 (by volume), and 5 mM genipin – the highest concentrations of each of the chemical crosslinking agents evaluated).

Table 1 lists the respective amide peaks of RpCol before and after the addition of the specified chemical crosslinking agents. Various concentrations and ratios of chemical crosslinking agents were investigated to determine if changing these aspects would lead to changes in the structure of RpCol, and amide peak wavenumber frequencies were evaluated to determine if the chemical groups of RpCol are comparable to that of mammalian collagen. The peak observed at Amide A, typically ranging from ∼3,300 to 3440 cm^−1^, is caused by N-H stretching vibrations coupled with hydrogen bonding (Felix et al., 2019; Paradiso et al., 2019). However, the exact wavenumber of this peak can be subject to variability, potentially shifting to lower frequencies due to N-H groups of collagens being involved in hydrogen bonding, which helps to hold the triple helical structures together within the collagen molecule (Felix et al., 2019). There is also potential interference from O-H stretching vibrations in the region 3,000–3,600 cm^−1^ (Reay et al., 2022; Reay et al., 2023). Despite freeze-drying samples to minimise the interference of water, variation in this region was reflected in the results observed (Table 1). A range of Amide A peaks from 3,261 to 3,353 cm^−1^ was denoted for uncrosslinked RpCol and RpCol crosslinked with EDC/sNHS 5% 1:1, respectively, potentially due to the aforementioned reasons that can generate differences in FTIR spectra within this specific range. Amide I, II, and III peaks relate to the structural conformation of collagen and normally range from 1,600 to 1,700 cm^−1^, 1,510–1,580 cm^−1^, and 1,200–1,300 cm^−1^, respectively, in mammalian collagen (Belbachir et al., 2009; de Campos Vidal and Mello, 2011; Riaz et al., 2018; Felix et al., 2019; Paradiso et al., 2019). The Amide I peak occurs because of C=O stretching vibrations/hydrogen bonding coupled with COO^-^. Potential shifts in wavenumber frequency between uncrosslinked and crosslinked collagen indicate that the C=O bond in collagen is affected by the formation of bonds between carbonyl and amine groups (Felix et al., 2019). The Amide I peak was observed in all the RpCol conditions investigated and fell within the expected range, typically occurring ∼1,650 cm^−1^ (Figure 1B; Table 1). The Amide II peak results from N-H bend coupled with C-N stretching vibrations, CH_2_ bend/wag, and COO^−^ symmetrical stretching vibrations (Felix et al., 2019; Paradiso et al., 2019). Again, the Amide II peak was present in all conditions investigated, most commonly occurring at 1,560 cm^−1^ (Figure 1B; Table 1). The presence of these Amide I and II peaks indicate a high molecular order of RpCol with hydrogen bonding present, thus providing stability to the triple helical structure. An Amide III peak, from N-H bend coupled with C-N stretching vibrations and C-O stretching vibrations, suggests the triple helical structure of RpCol (Felix et al., 2019; Paradiso et al., 2019) and was present in all observed conditions (Figure 1B; Table 1). Variations in wavenumber frequencies of Amide III peaks outside of the expected range can indicate that crosslinking has affected the collagen structure (Felix et al., 2019). This can be demonstrated by the results that we observed, whereby different compositions of chemical crosslinking agents induced differences in Amide III peak frequencies, ranging from 1,239 to 1,334 cm^−1^ for 4-arm PEG-SCM 4% and EDC/sNHS 10% 2:1, respectively (Table 1). The peak observed at 1,450 cm^−1^ is attributed to CH_3_ asymmetric bending vibration (Li et al., 2023). The ratio of the intensity of the Amide III peak to that of the peak at 1,450 cm^−1^ (Amide III/A_1450_ ratio) provides an indication of the integrity of the triple helical structure, with a ratio of far less than 1.00 showing the loss of this structure (Li et al., 2023). The Amide III/A_1450_ ratio of uncrosslinked RpCol was 1.02, indicating the integrity of the triple helical structure (Table 1). Interestingly, the Amide III/A_1450_ ratio varied slightly following the addition of chemical crosslinking agents, however, for most conditions this ratio was close to 1.00, suggesting preservation of structure (Table 1). The slight exceptions to this were when RpCol was crosslinked with 4-arm PEG-SCM 8% and 4-arm PEG-SCM 16%, where the Amide III/A_1450_ ratio decreased to slightly less than 1.00 (0.81 and 0.80, respectively) (Table 1).

**TABLE 1:** FTIR-ATR characteristic peaks and Amide III/A_1450_ ratio. Assigned peaks of Amide A, I, II, and III, and Amide III/A_1450_ ratio of RpCol before (uncrosslinked RpCol) and after crosslinking with chemical crosslinking agents (4%, 8%, and 16% 4- and 8-arm PEG-SCM, 1%, 5%, and 10% EDC/NHS and EDC/sNHS 1:1 and 2:1, and 1 mM, 2.5 mM, and 5 mM genipin) to form hydrogels.

Sample	Wavenumber (cm^−1^)				
	Amide A	Amide I	Amide II	Amide III	Amide III/A_1450_ ratio
Uncrosslinked RpCol	3,261	1,655	1,541	1,262	1.02
4-arm PEG-SCM 4%	3,284	1,653	1,560	1,264	1.00
4-arm PEG-SCM 8%	3,329	1,647	1,560	1,239	0.81
4-arm PEG-SCM 16%	3,311	1,647	1,560	1,241	0.80
8-arm PEG-SCM 4%	3,312	1,655	1,560	1,295	1.04
8-arm PEG-SCM 8%	3,315	1,648	1,560	1,314	1.06
8-arm PEG-SCM 16%	3,299	1,648	1,551	1,314	1.07
EDC/NHS 1% 1:1	3,299	1,636	1,560	1,314	1.07
EDC/NHS 1% 2:1	3,286	1,638	1,551	1,314	1.08
EDC/NHS 5% 1:1	3,297	1,638	1,560	1,306	1.08
EDC/NHS 5% 2:1	3,323	1,638	1,560	1,312	1.06
EDC/NHS 10% 1:1	3,301	1,638	1,560	1,306	1.09
EDC/NHS 10% 2:1	3,293	1,636	1,560	1,306	1.10
EDC/sNHS 1% 1:1	3,301	1,648	1,560	1,316	1.07
EDC/sNHS 1% 2:1	3,301	1,653	1,560	1,319	1.07
EDC/sNHS 5% 1:1	3,353	1,648	1,560	1,286	0.98
EDC/sNHS 5% 2:1	3,330	1,647	1,560	1,310	1.07
EDC/sNHS 10% 1:1	3,330	1,647	1,560	1,333	1.17
EDC/sNHS 10% 2:1	3,323	1,647	1,560	1,334	1.14
Genipin 1 mM	3,284	1,647	1,545	1,297	1.08
Genipin 2.5 mM	3,288	1,655	1,551	1,262	1.03
Genipin 5 mM	3,284	1,655	1,560	1,264	0.97

Overall, Amide A, I, II, and III peaks, reflecting peptide bond vibrations, were observed in all the conditions of RpCol. It is worth noting that the addition of chemical crosslinking agents, and varying their compositions, resulted in wavenumber frequency shifts in all the amide peaks investigated, suggesting a potential change in the molecular organisation and therefore impact on the stability of RpCol following crosslinking.

### 2.2 RpCol hydrogel stability and hydrolytic degradation

**FIGURE 2:**
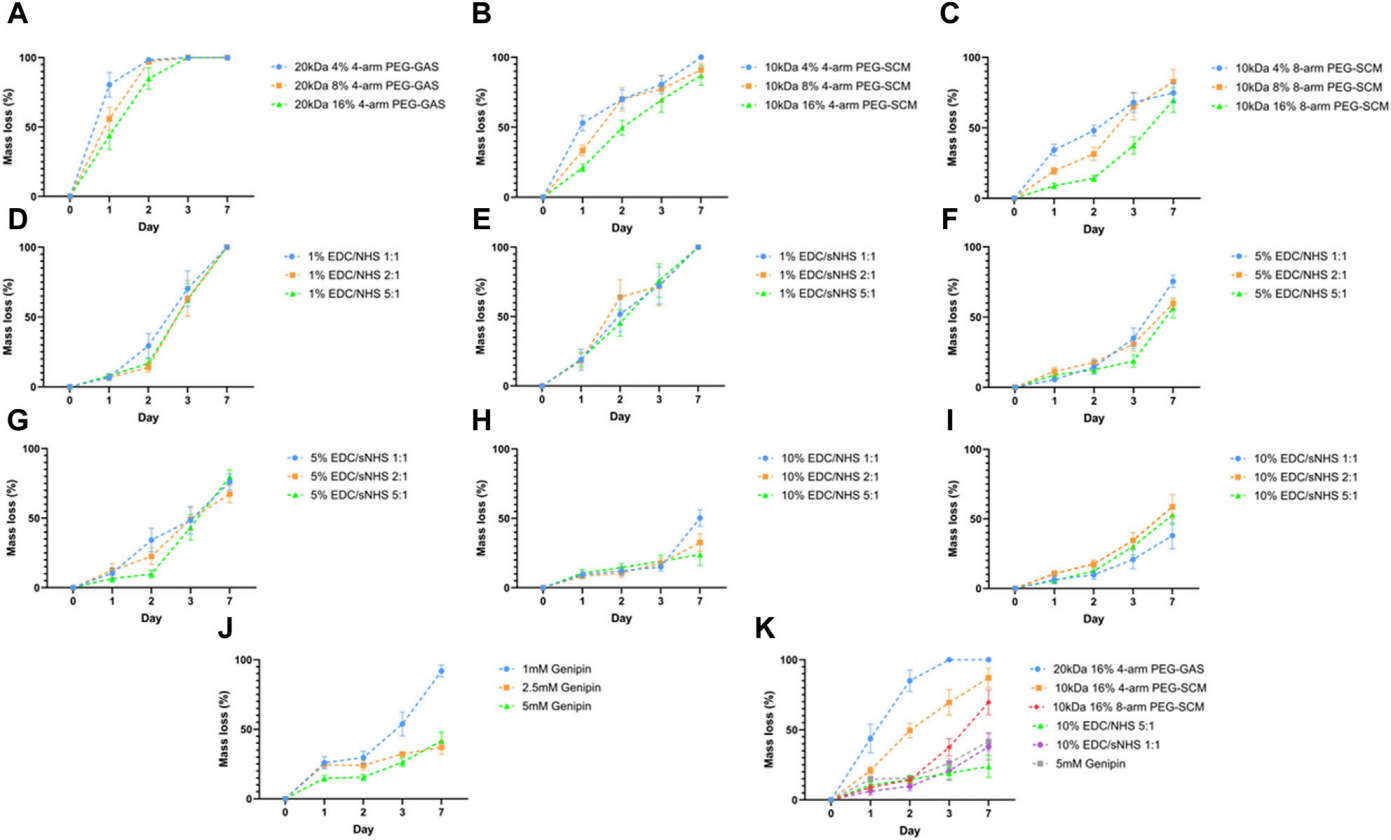
Hydrolytic degradation of RpCol hydrogels formed with chemical crosslinking agents. **(A–C)** Multi-arm functionalised PEG derivatives at concentrations of 4%, 8%, and 16%; **(A)** 20 kDa 4-arm PEG-GAS, **(B)** 10 kDa 4-arm PEG-SCM, **(C)** 10 kDa 8-arm PEG-SCM. **(D–I)** EDC/NHS or EDC/sNHS at ratios 1:1, 2:1, and 5:1 at concentrations of **(D)** 1% EDC/NHS, **(E)** 1% EDC/sNHS, **(F)** 5% EDC/NHS, **(G)** 5% EDC/sNHS, **(H)** 10% EDC/NHS, **(I)** 10% EDC/sNHS. **(J)** Different concentrations of genipin; 1, 2.5, and 5 mM. **(K)** Most stable formulation of each crosslinker; 20 kDa 16% 4-arm PEG-GAS, 10 kDa 16% 4-arm PEG-SCM, 10 kDa 16% 8-arm PEG-SCM, 10% EDC/NHS 5:1, 10% EDC/sNHS 1:1, and 5 mM genipin. Data presented as mean ± standard error of the mean of three independent experiments performed in triplicate.

To assess the potential of RpCol hydrogels to be utilised in regenerative medicine applications, RpCol hydrogels need to be stable enough at physiological conditions for a sustained period. Depending on the application, prolonged stability of RpCol hydrogels may be essential to ensure maximum therapeutic efficacy. We found that uncrosslinked RpCol did not form stable hydrogels, which concurs with the literature (Riacci et al., 2021). Since the denaturation of jellyfish collagen is reported to be at lower temperatures than mammalian collagen (Oliveira et al., 2021; Smith et al., 2023), we investigated the degradation profiles of RpCol hydrogels formed with a variety of chemical crosslinking agents (Figure 2). The concentration of RpCol chosen was 4.46 mg/mL, which is the concentration following the addition of neutralising buffer to 5 mg/mL RpCol, as this concentration was viscous enough to allow initial hydrogel formation, yet not too viscous to encounter manipulability issues.

Initial hydrolytic degradation experiments were conducted using 20 kDa 4-arm PEG-GAS, provided by Jellagen Limited as part of a now discontinued kit, as the chemical crosslinking agent. The concentrations of the NHS ester-terminating functionalised PEGs chosen throughout all the RpCol hydrogel characterisations were 4%, 8%, and 16%, as these concentrations were successfully used for the formation of bovine collagen hydrogels (Fernandes-Cunha et al., 2020). RpCol hydrogels crosslinked with 20 kDa 4-arm PEG-GAS exhibited rapid degradation at all concentrations tested, with all hydrogels fully degraded within 3 days, and all ∼50% degraded within 1 day (Figure 2A). As these functionalised PEGs degrade by hydrolysis, it was hypothesised that the use of a lower MW would provide a denser hydrogel structure and thus reduce the rate of hydrogel degradation. However, hydrogels again exhibited rapid degradation with the use of 5 kDa PEG-GAS as the chemical crosslinking agent, with all concentrations fully degraded within 3 days (data not shown). RpCol hydrogels formed with PEG-GAS may have other potential uses, for example in drug delivery where rapid degradation may be desirable; however, these hydrogels were deemed unsuitable to assess their potential for use in regenerative medicine. RpCol hydrogel stability needed to be increased to allow their effects on cells to be determined, as many cells, including MSCs (widely utilised in regenerative medicine), produce matrix metalloproteinases (MMPs), enzymes capable of further degrading collagen (Mazzeo et al., 2019). Upon examination of the structures, it was found that connecting the main body of the PEG molecule and the NHS molecule in PEG-GAS is a secondary amide, providing a potential site of hydrolysis. PEG-SCM does not contain any extra groups in this region, thus it was hypothesised that less hydrolysis would occur with the use of PEG-SCM and therefore the rate of hydrogel degradation would reduce. It was also hypothesised that using PEG-SCM with an increased number of arms would increase hydrogel stability, providing more potential crosslinking sites. RpCol hydrogel stability did indeed increase with the use of both 4- and 8-arm 10 kDa PEG-SCM as the chemical crosslinking agent compared to PEG-GAS, as well as increase with the use of these PEGs with an increased number of arms (Figures 2B, C). 16% 4- and 8-arm PEG-SCM hydrogels took significantly longer to degrade than 4% PEG-SCM hydrogels (*p* < 0.01** and *p* < 0.0001****, respectively). Furthermore, 16% 8-arm PEG-SCM hydrogels took significantly longer to degrade than 16% 4-arm PEG-SCM hydrogels (*p* < 0.001***), exhibiting only ∼37.5% mass loss after 3 days, therefore demonstrating that 8-arm PEG-SCM RpCol hydrogels are more stable than 4-arm PEG-SCM RpCol hydrogels (Figures 2B, C).

EDC, with or without NHS or sNHS, is a popular choice for crosslinking of collagen-based biomaterials. However, the concentration of EDC typically used is ∼1% (w/v or v/v) (Widdowson et al., 2018; Flaig et al., 2020; Ahmed et al., 2021), as higher concentrations have been shown to exhibit cytotoxicity (Thoreson et al., 2015). Thus, we initially investigated an EDC concentration of 1% (v/v) (Figures 2D, E). All RpCol hydrogels crosslinked with 1% EDC at all ratios of EDC/NHS and EDC/sNHS (1:1, 2:1, and 5:1) degraded rapidly (Figures 2D, E, respectively), demonstrating ∼75% mass loss by day 3. To attempt to increase RpCol hydrogel stability to a suitable degradation rate for potential use in regenerative medicine, we increased the EDC concentration to 5% and 10% (Figures 2F–I, respectively). For all ratios assessed, RpCol hydrogels crosslinked with both 5% and 10% EDC demonstrated increased stability, with all hydrogels losing 50% or less of their mass by day 3. The use of EDC/NHS 10% 5:1 resulted in a mass loss of only ∼25% after 3 days (Figure 2H).

Like the reported result of RpCol hydrogels crosslinked with genipin in the literature (Riacci et al., 2021), we observed that 1 mM genipin did not produce stable hydrogels. However, crosslinking RpCol with both 2.5 mM and 5 mM genipin resulted in stable hydrogels, displaying only ∼30% mass loss after 3 days, significantly less than RpCol hydrogels crosslinked with 1 mM genipin (*P* < 0.0001****) (Figure 2J). Figure 2K illustrates the compositions of each of the chemical crosslinking agents investigated that resulted in the least mass loss of RpCol hydrogels. It is evident that a variety of RpCol hydrogel degradation rates can be achieved by utilising different chemical crosslinking agents, ranging from 100% mass loss by day 3 when using 20 kDa PEG-GAS to ∼25% mass loss with the use of EDC/NHS 10% 5:1. Therefore, RpCol hydrogels may have the potential to be used in a wide range of applications, where the desired degradation rate can inform the choice of crosslinking agent.

### 2.3 Morphological properties of RpCol hydrogels

As we are investigating the potential of RpCol hydrogels to be utilised in regenerative medicine, where there will be interactions with cells and molecules in a three-dimensional (3D) environment, it is important to first understand the microstructures of RpCol hydrogels formed with the different chemical crosslinking agents. The morphological structure of RpCol hydrogels is also an important aspect to consider if potential applications require the incorporation of further therapeutic cargo for example. Pore size has been shown to affect nutrient and gas diffusion, as well as cell adhesion, proliferation, and migration (Murphy et al., 2010; Matsiko et al., 2015). Murphy et al. seeded an osteoblastic cell line onto a collagen scaffold and determined that the largest pore size, with a diameter of ∼325 µm, maintained a significantly higher cell number than with smaller pore sizes. However, they also demonstrated that the scaffolds with the smallest pores facilitated the greatest cell attachment, most likely due to a greater number of available binding motifs (Murphy et al., 2010). Pores of ∼300 µm have also been shown to significantly increase chondrogenic expression of MSCs in comparison to smaller pores, indicating that pore size can influence tissue regeneration (Matsiko et al., 2015).

**FIGURE 3:**
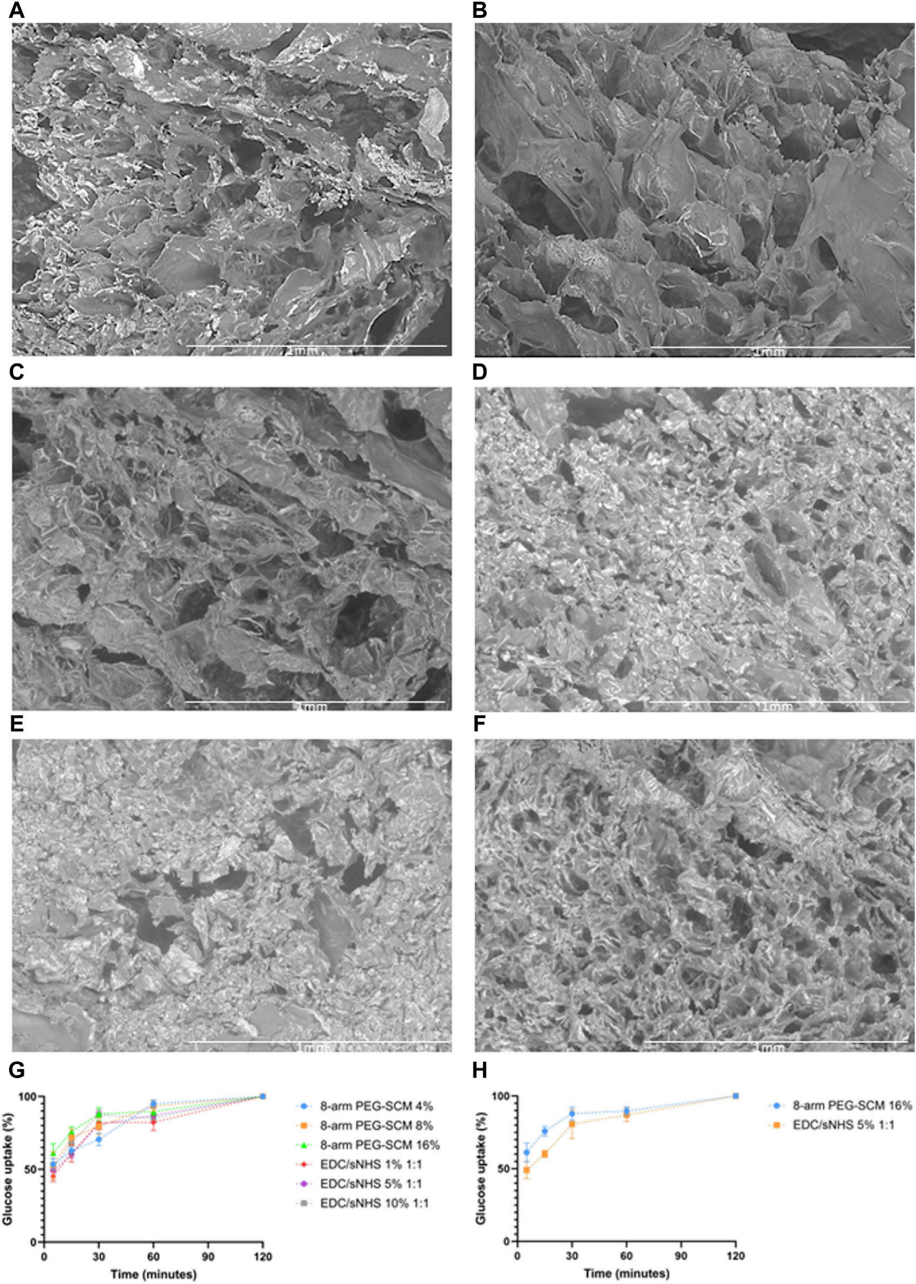
**(A–F)** Representative SEM images at × 100 magnification of the cross-sections of RpCol before and after the addition of chemical crosslinking agents; **(A)** uncrosslinked RpCol, **(B)** 4-arm PEG-SCM, **(C)** 8-arm PEG-SCM, **(D)** EDC/NHS, **(E)** EDC/sNHS, and **(F)** genipin, to form hydrogels. **(G, H)** Glucose uptake of RpCol hydrogels formed with selected chemical crosslinking agents; **(G)** 4%, 8%, and 16% 8-arm PEG-SCM and 1%, 5%, and 10% EDC/sNHS 1:1, and **(H)** 16% 8-arm PEG-SCM and 5% EDC/sNHS 1:1. Data presented as mean ± standard error of the mean of two independent experiments performed in triplicate.

Overall, a wide range of pore structures with varying pore diameter distributions of RpCol can be observed in Figures 3A–F and Table 2. Figures 3A–F displays representative cross-sectional scanning electron microscopy (SEM) images of uncrosslinked RpCol (Figure 3A), and RpCol hydrogels crosslinked with the highest concentrations of each chemical crosslinking agent (16% 4- and 8-arm PEG-SCM, 10% EDC/NHS and EDC/sNHS 1:1, and 5 mM genipin); 4-arm PEG-SCM (Figure 3B), 8-arm PEG-SCM (Figure 3C), EDC/NHS (Figure 3D), EDC/sNHS (Figure 3E), and genipin (Figure 3F). All conditions demonstrated a microporous RpCol hydrogel structure (Figures 3A–F; Table 2). Based on all the results from all the characterisations performed, we decided to proceed with 8-arm PEG-SCM and EDC/sNHS as the chemical crosslinking agents of choice to form RpCol hydrogels to test their effects on cells. 16% 8-arm PEG-SCM and EDC/sNHS 5% 1:1 were the concentrations of choice, as these were deemed the optimum conditions to form stable RpCol hydrogels while aiming to maintain a favourable environment for cells. The mean pore diameters of RpCol hydrogels formed with the chemical crosslinking agents chosen to perform cellular experiments were 100 and 113 μm for 16% 8-arm PEG-SCM and EDC/sNHS 5% 1:1, respectively (Table 2). While both values are below the 300–325 μm range mentioned, as RpCol has demonstrated reduced cell adhesion (including by MSCs), RpCol hydrogels possessing smaller pore diameters may facilitate greater cell adhesion (Murphy et al., 2010; Smith et al., 2023).

**TABLE 2:** Pore diameters. Minimum, maximum, mean, and standard deviation of pore diameters (μm to 3 significant figures) of RpCol before (uncrosslinked RpCol) and after crosslinking with chemical crosslinking agents (4%, 8%, and 16% 4- and 8-arm PEG-SCM, 1%, 5%, and 10% EDC/NHS and EDC/sNHS 1:1, and 1 mM, 2.5 mM, and 5 mM genipin) to form hydrogels.

Sample	Minimum (µm)	Maximum (µm)	Mean (µm)	Standard deviation (µm)
Uncrosslinked RpCol	32.0	120	62.0	21.0
4-arm PEG-SCM 4%	3.00	528	125	93.0
4-arm PEG-SCM 8%	23.0	106	46.0	18.0
4-arm PEG-SCM 16%	59.0	400	143	56.0
8-arm PEG-SCM 4%	17.0	101	42.0	15.0
8-arm PEG-SCM 8%	38.0	241	108	54.0
8-arm PEG-SCM 16%	24.0	463	100	109
EDC/NHS 1% 1:1	24.0	112	49.0	18.0
EDC/NHS 5% 1:1	88.0	509	255	115
EDC/NHS 10% 1:1	54.0	338	154	68.0
EDC/sNHS 1% 1:1	23.0	168	62.0	26.0
EDC/sNHS 5% 1:1	38.0	285	113	58.0
EDC/sNHS 10% 1:1	71.0	736	234	146
Genipin 1 mM	16.0	160	44.0	24.0
Genipin 2.5 mM	29.0	348	120	80.0
Genipin 5 mM	34.0	110	61.0	18.0

2-NBDG (2-(*N*-(7-Nitrobenz-2-oxa-1,3-diazol-4-yl)Amino)-2-Deoxyglucose) is a fluorescent glucose analogue typically used to determine the glucose diffusion rate of a material. The pore size of a hydrogel and its diffusion rate of molecules are likely to be closely related, with the fluid dynamics resembling that of Fick’s Law of Diffusion. We observed no significant difference of glucose uptake of RpCol hydrogels between any of the conditions of 8-arm PEG-SCM and EDC/sNHS and therefore no significant difference for the concentrations selected for cellular experiments (16% 8-arm PEG-SCM and EDC/sNHS 5% 1:1) (Figures 3G, H, respectively). Initial timepoints demonstrated a rapid rate of diffusion, with RpCol hydrogels reaching ∼50% glucose uptake by 5 min. By 30 min, the rate of glucose uptake had slowed, maintaining a gradual upward plateau until 120 min, where 100% of glucose uptake had occurred.

Thus, these results suggest that the pore size of RpCol hydrogels had no effect on glucose uptake. Indeed, a potential explanation of the variation in pore sizes observed is that the freeze-drying process to prepare the RpCol hydrogels for SEM imaging may not have sufficiently preserved the hydrogel pore structures [as this technique can result in surface collapse for example (Reay et al., 2022)] and that there is in fact less variation than observed. Overall, the glucose uptake rate of RpCol hydrogels was rapid, comparable to a study in our group using a collagen-alginate-fibrin (CAF) scaffold (da Conceicao Ribeiro et al., 2018), indicating that RpCol hydrogels may be able to maintain the viability of encapsulated cells and thus be utilised in a wide range of applications.

### 2.4 Rheological analyses of formulated RpCol hydrogels

**FIGURE 4:**
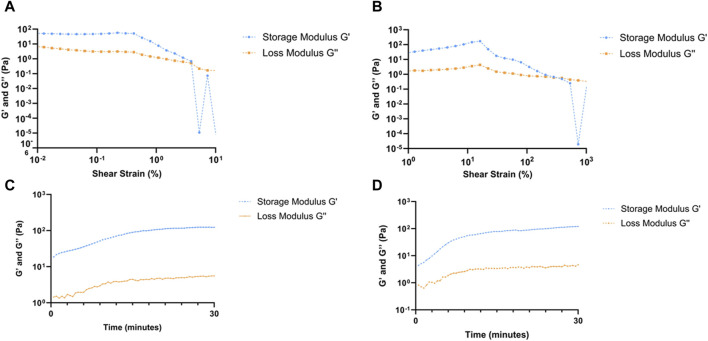
Rheological analyses of RpCol hydrogels formed with selected chemical crosslinking agents. **(A, B)** amplitude sweeps of RpCol hydrogels crosslinked with; **(A)** 16% 8-arm PEG-SCM, and **(B)** EDC/sNHS 5% 1:1. **(C, D)** time sweeps of RpCol hydrogels crosslinked with; **(C)** 16% 8-arm PEG-SCM, and **(D)** EDC/sNHS 5% 1:1.

Rheological analyses were conducted to assess the mechanical properties of RpCol hydrogels formed with the various chemical crosslinking agents described and thus evaluate their suitability for potential use depending on the desired application (Supplementary Table S1; Supplementary Table S2; Figure 4). Rheological assessments allow the measurement of the storage (elastic) modulus (G’) and the loss (viscous) modulus (G”) of a material, with these reflecting the elasticity upon deformation and how the material flows upon deformation, respectively (Stojkov et al., 2021). Amplitude sweeps, which use back and forth oscillations at an increasing amplitude, examine the degree of shear stress a material can undergo before deforming. Time sweeps use a set oscillation to investigate the change in structure of a material over time. In the case of hydrogels, time sweeps can also allow the gelation time to be determined (Stojkov et al., 2021).

Due to the unstable nature of uncrosslinked RpCol, rheological analyses were unable to be conducted on uncrosslinked RpCol hydrogels. (Supplementary Table S1; Supplementary Table S2) illustrates the values generated from crosslinked RpCol hydrogels from their amplitude and time sweeps, respectively. Figures 4A–D demonstrate the respective amplitude and time sweeps of the conditions chosen for cellular experiments, 16% 8-arm PEG-SCM and EDC/sNHS 5% 1:1, respectively.

Amplitude sweeps exhibit a linear viscoelastic region (LVR), followed by a yield point and a crossover point. The LVR defines the range whereby the moduli are unaffected by an increase in amplitude, where a longer LVR indicates that a greater shear strain is needed to permanently deform a material. The yield point occurs at the end of the LVR, representing the start of plastic deformation, with the crossover point of the moduli signifying complete deformation of a material (Stojkov et al., 2021). For both RpCol hydrogels crosslinked with 16% 8-arm PEG-SCM and EDC/sNHS 5% 1:1, the LVR ranged from 1.0% to 42.0% shear strain, with the yield points occurring at a shear strain of 42.0% (Figures 4A, B, respectively), both relatively high compared to RpCol hydrogels formed with different compositions of chemical crosslinking agents (Supplementary Table S1). The crossover points of these PEG- and EDC-crosslinked RpCol hydrogels occurred at ∼400% and ∼550% shear strain, respectively, again relatively high compared to other crosslinked RpCol hydrogels (Supplementary Table S1).

Interestingly, as also observed in the amplitude sweeps of all RpCol hydrogels (Supplementary Table S1; Figures 4A, B), G’ appeared above G” from the first timepoint recorded (30 s) in the time sweeps of all RpCol hydrogels evaluated (Supplementary Table S2; Figures 4C, D), indicating that these RpCol hydrogels already exhibited a gel-like (elastic) behaviour (Riacci et al., 2021; Stojkov et al., 2021). This finding agrees with the study conducted on RpCol hydrogels crosslinked with genipin, which also found G’ to be above G” at the first timepoint recorded for all concentrations of crosslinker assessed (Riacci et al., 2021). No crossover points and therefore no gelation times could be determined from these rheological assessments. This suggests that either the crosslinking of RpCol hydrogels was rapid and gelation had already occurred before the first 30 s timepoint, or that the RpCol solution (prior to crosslinking) already exhibits this elastic behaviour.

Time sweeps were conducted over 30 min immediately following the addition of a chemical crosslinking agent to RpCol solution, allowing us to observe the initial changes in the structure of RpCol following crosslinker addition. All time sweeps exhibited gradual increases in G’ and G” over time, indicating an increase in the stiffness of RpCol hydrogels, most likely because of crosslinking occurring (Stojkov et al., 2021). The first and last G’ values for 16% 8-arm PEG-SCM- and EDC/sNHS 5% 1:1-crosslinked RpCol hydrogels were 18.6 Pa and 123 Pa, and 4.37 Pa and 121 Pa, respectively (Supplementary Table S2; Figures 4C, D). The initial value of G’ was greater in the PEG-crosslinked RpCol hydrogels compared to the EDC-crosslinked RpCol hydrogels, suggesting a greater rate of crosslinking with the use of PEG-SCM as the chemical crosslinking agent. Intriguingly, the final G’ values (after 30 min) of all crosslinked RpCol hydrogels were significantly less than the 11–30 kPa range that has been shown to induce osteogenesis and differentiation of MSCs (Huebsch et al., 2010), perhaps suggesting that RpCol hydrogels are unlikely to induce MSC differentiation and thus likely to maintain any potential immunomodulatory properties of MSCs. Surprisingly, the final G’ values of RpCol hydrogels demonstrate that crosslinking with 4-arm PEG-SCM produced the stiffest RpCol hydrogels, a property that was not reflected in the degradation kinetics of the hydrogels, where 4-arm PEG-SCM-crosslinked RpCol hydrogels were relatively unstable compared to other crosslinked RpCol hydrogels (Figure 3). This highlights the importance of the choice of chemical crosslinking agent depending on the intended application and demonstrates the adaptability of RpCol hydrogels for a variety of applications, as their properties can be tuned to elicit the desired effects through the addition of different crosslinking agents.

### 2.5 Endotoxin mechanism and endotoxin quantification of RpCol

**FIGURE 5:**
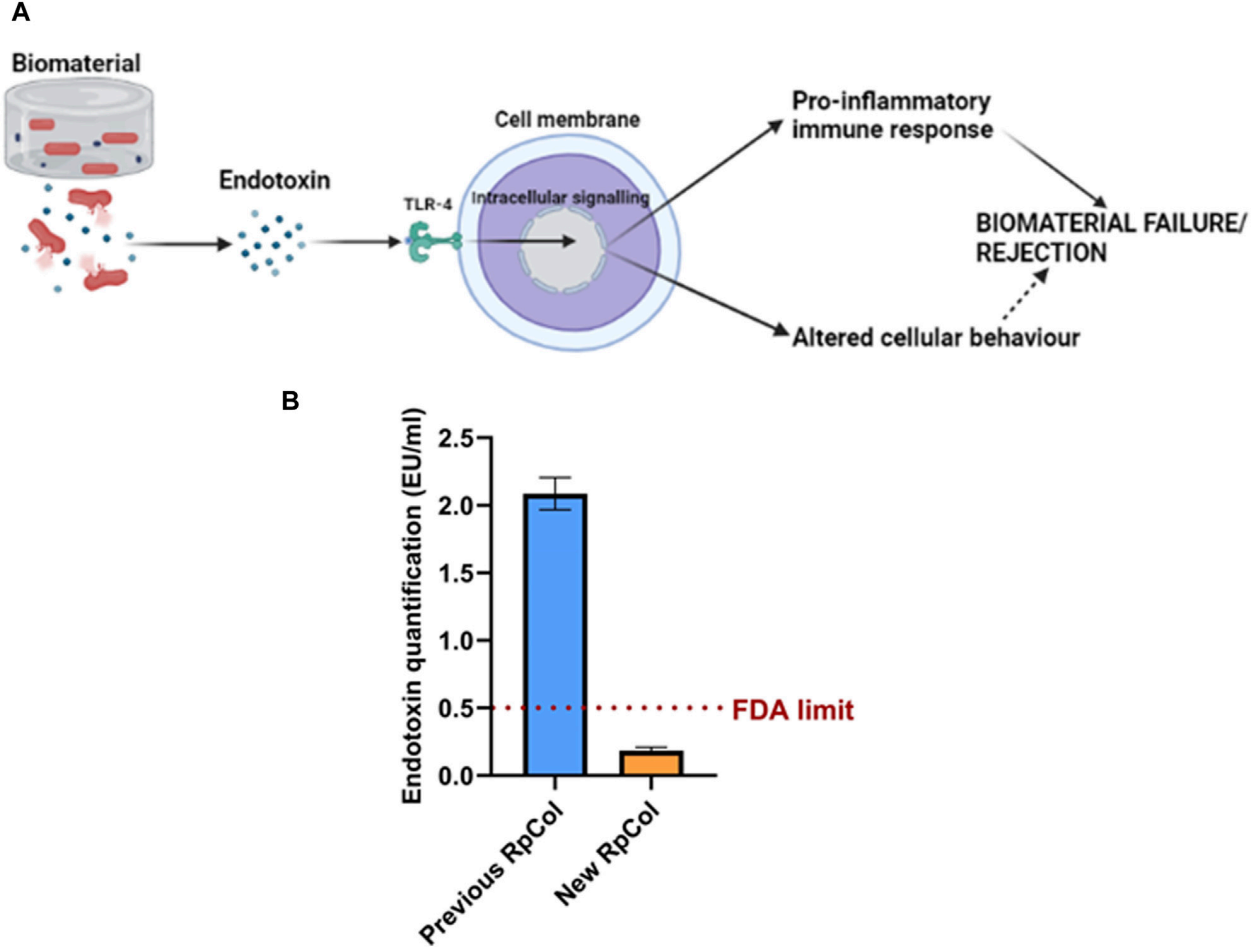
**(A)** Mechanism of endotoxin. Created in BioRender.com. **(B)** Endotoxin concentration (EU/mL) of previous RpCol and new RpCol. Data presented as mean ± standard error of the mean of two independent experiments performed in triplicate.

The contamination of biomaterials with bacteria, and therefore endotoxin, is ubiquitous, as endotoxin is found in the outer membrane of Gram-negative bacteria (Salthouse et al., 2023). The cellular mechanism of endotoxin is depicted in Figure 5A. Extremely small concentrations of endotoxin can elicit potent pro-inflammatory host immune responses (through the Toll-like receptor (TLR)-4 pathway), which can significantly affect the performance of biomaterials and can lead to biomaterial failure/rejection (Heinrich et al., 2023; Reay et al., 2023; Salthouse et al., 2023). Endotoxin has also been shown to influence cellular behaviour; inhibiting bone formation, cartilage formation, and dermal wound healing for example (Xu et al., 2018; Wang L. et al., 2020; Groen et al., 2020; Zhao et al., 2020), therefore the presence of endotoxin may lead to incorrect conclusions being made about potential biomaterial-based therapies. However, despite its importance, the issue of endotoxin contamination of biomaterials is not widely known/addressed in the biomaterials community, with the endotoxin levels of biomaterials rarely being considered/reported (Salthouse et al., 2023). No studies have quantified the endotoxin levels of jellyfish collagen and therefore of RpCol, thus, this is the first biological characterisation that we conducted. Endotoxin levels of biomaterials must be below the FDA limit (0.5 endotoxin units (EU)/ml) to allow their use *in vivo* (Salthouse et al., 2023).

Previous RpCol (now discontinued) presented endotoxin levels above regulatory limits (>0.5 EU/mL) (Figure 5B). It is worth nothing that the upper detection limit for this assay is 1 EU/mL and therefore the actual endotoxin concentration may be higher than the observed concentration. However, following an endotoxin removal process (by Jellagen Limited), the endotoxin levels of RpCol were below regulatory limits (<0.5 EU/mL) (Figure 5B), thus allowing the potential use of RpCol *in vivo*. The low endotoxin levels of RpCol could also allow RpCol hydrogels to be utilised in *in vitro* models, as cellular behaviour is unlikely to be affected by endotoxin.

### 2.6 Effects of encapsulating TERT-hMSCs within RpCol hydrogels and anti-inflammatory cytokine production

**FIGURE 6:**
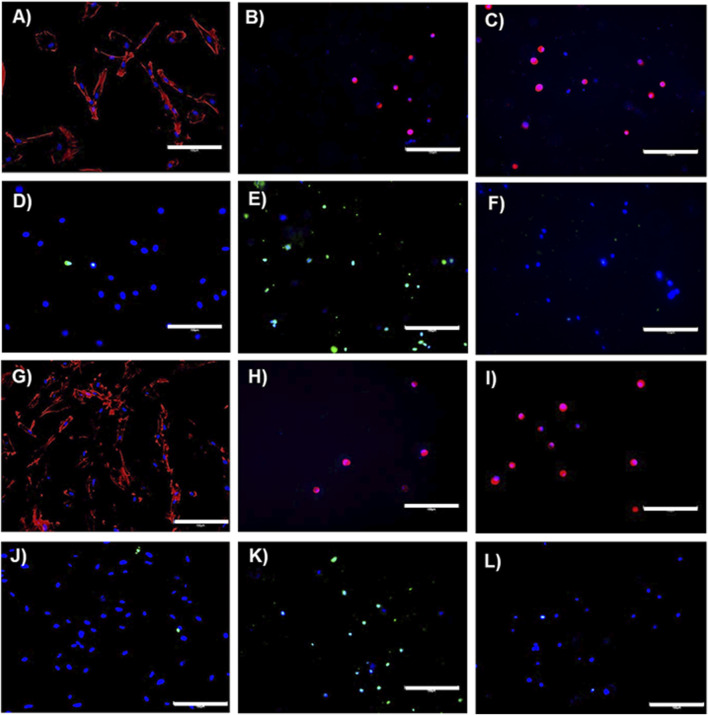
Immunostaining. Representative images on day 1 **(A–F)** and day 3 **(G–L)** of the morphology **(A–C)** and **(G–I)** and viability **(D–F)** and **(J–L)** of TERT-hMSCs encapsulated within RpCol hydrogels. **(A, D, G, J)** = controls (cells cultured on tissue culture plastic). **(B, E, H, K)** = 5% EDC/sNHS 1:1-crosslinked RpCol hydrogels. **(C, F, I, L)** = 16% 8-arm PEG-SCM-crosslinked RpCol hydrogels. Scale bars = 150 µm.

MSCs are widely utilised in regenerative medicine due to their differentiating ability and regenerative and immunomodulatory properties to promote tissue regeneration (Salthouse et al., 2023). However, the therapeutic efficacy of MSCs can be significantly affected by their microenvironment. Ways of utilising biomaterials to increase the success of MSC therapies are currently being explored, including encapsulation within hydrogels/scaffolds to provide a favourable microenvironment for MSCs to maintain their desired effects for a prolonged period (Salthouse et al., 2023). We therefore investigated the effects of encapsulating TERT-hMSCs within RpCol hydrogels on cellular morphology, viability, metabolic activity, and anti-inflammatory cytokine production. If RpCol hydrogels can provide a favourable environment to sustain the properties of MSCs, they have great potential to be utilised in a wide range of applications. Figures 6A, D, G, J display representative images of the morphology (Figures 6A, G) and viability (Figures 6D, J) of TERT-hMSCs cultured on tissue culture plastic (control cells) after 1 day (Figures 6A, D) and 3 days (Figures 6G, J). Imaging of EDC/sNHS 5% 1:1-crosslinked RpCol hydrogels showed that most of these encapsulated MSCs were dead on both day 1 and 3 (Figures 6E, K, respectively), and exhibited a rounded morphology (Figures 6B, H, respectively).

**FIGURE 7:**
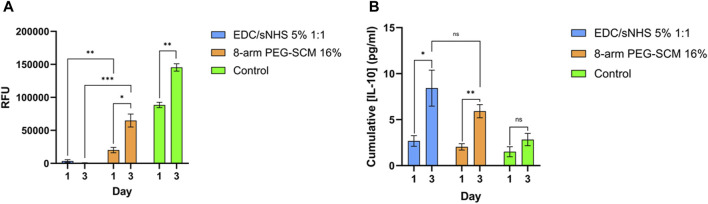
Metabolic activity and anti-inflammatory cytokine production. **(A)** metabolic activity of TERT-hMSCs encapsulated within RpCol hydrogels formed with selected chemical crosslinking agents (EDC/sNHS 5% 1:1 and 8-arm PEG-SCM 16%). **(B)** IL-10 production of TERT-hMSCs encapsulated within RpCol hydrogels formed with selected chemical crosslinking agents (EDC/sNHS 5% 1:1 and 8-arm PEG-SCM 16%). Data presented as mean ± standard error of the mean of two independent experiments performed in triplicate. Asterix represent degree of significance; *p* < 0.05*, *p* < 0.01**, *p* < 0.001***, *p* < 0.0001****. Paired t-tests were used to analyse the same chemical crosslinking agent between different days. Unpaired t-tests were used to analyse different chemical crosslinking agents between the same day. RFU = relative fluorescence units.

The metabolic activity of TERT-hMSCs encapsulated within these EDC-crosslinked RpCol hydrogels was also very low (Figure 7A), further indicating cell death. As EDC is generally used at concentrations of ∼1% (as it is indicated that higher EDC concentrations are cytotoxic) (Thoreson et al., 2015; Widdowson et al., 2018; Flaig et al., 2020; Ahmed et al., 2021), and as we used an EDC concentration of 5% (to produce stable enough hydrogels), it is understandable that cytotoxic effects were elicited. There is, however, limited explanation in the literature on the exact mechanism causing this EDC-mediated cytotoxicity. Dong et al demonstrated that at higher concentrations of EDC, more of the waste product urea is produced, which causes cell cycle changes and apoptosis (Dong et al., 2013). In contrast, RpCol hydrogels crosslinked with 16% 8-arm PEG-SCM did not cause cell death (Figures 6F, L), and there was a significant increase in metabolic activity of TERT-hMSCs (*p* < 0.05*) between day 1 and 3 (Figure 7A). This indicates that 16% 8-arm PEG-SCM-crosslinked RpCol hydrogels are non-cytotoxic and allow the maintenance of normal cellular processes. TERT-hMSCs, however, also exhibited a rounded morphology when encapsulated in these PEG-crosslinked RpCol hydrogels (Figures 6C, I), potentially due to the stiffness of the hydrogels, and the fact that the MSCs were housed in a 3D environment. Overall, sufficient nutrient diffusion must have occurred to maintain (and increase) the normal cellular processes of the TERT-hMSCs, therefore demonstrating the potential of 16% 8-arm PEG-SCM-crosslinked RpCol hydrogels to be utilised in a wide range of regenerative medicine applications.

The use of biomaterials for regenerative medicine applications continues to grow, however, the clinical uptake of a wide variety of biomaterials remains low, which is due to host immune responses towards biomaterials (Salthouse et al., 2023). A host immune response is required and has been shown to be essential for the success of a biomaterial, for example by mediating successful tissue regeneration and healing, however, it is the nature of the response that determines the success of a biomaterial, as an excessive response can ultimately result in biomaterial failure (Salthouse et al., 2023). MSCs have demonstrated immunomodulatory ability, with their secretome comprising a wide array of cytokines, chemokines, and growth factors that can modulate the functions of host immune cells, promoting a regenerative response (Salthouse et al., 2023).

Interleukin (IL)-10 is a potent anti-inflammatory cytokine, shown to be secreted by MSCs (Komai et al., 2018; Saxton et al., 2021), that can polarise macrophages (a pivotal cell in the host immune response to biomaterials) from a pro-inflammatory (or “M1”) phenotype to an anti-inflammatory (or “M2”) phenotype and thus promote tissue regeneration (Salthouse et al., 2023). It has also been suggested that RpCol elicits immunomodulatory effects, where RpCol scaffolds were shown to induce a long-term anti-inflammatory macrophage response and significant bone regeneration following implantation (Flaig et al., 2020). Thus, it was important to examine the cytokine production of the TERT-hMSCs encapsulated within RpCol hydrogels to elucidate the nature of the MSC response that would be elicited.

For both RpCol hydrogels crosslinked with EDC/sNHS 5% 1:1% and 16% 8-arm PEG-SCM, there was a significant increase in IL-10 production by encapsulated TERT-hMSCs between day 1 and 3 (Figure 7B). It is therefore unclear whether this response is due to a favourable or unfavourable environment, as our findings clearly indicate that these PEG-crosslinked RpCol hydrogels provide the former, whereas these EDC-crosslinked RpCol hydrogels provide the latter. It is interesting that the MSCs encapsulated within EDC/sNHS 5% 1:1-crosslinked RpCol hydrogels exhibited an increase in IL-10 production between day 1 and 3, as cell viability imaging and metabolic assays suggested that this concentration of EDC was cytotoxic and that most cells were dead. A potential explanation could be that the remaining cells continued to produce IL-10 until day 3 in response to the unfavourable environment. It is important to emphasise that EDC is a widely utilised crosslinking agent (Widdowson et al., 2018; Flaig et al., 2020; Ahmed et al., 2021), however, the high concentration that was required to form stable RpCol hydrogels in this study clearly rendered the use of EDC unsuitable for this purpose. RpCol has been shown to be associated with a downregulation of pathways related to inflammation (Bowen et al., 2022), which offers a possible explanation to the increase in IL-10 production observed.

Despite IL-10 being a potent anti-inflammatory cytokine and therefore the respective levels of IL-10 being a good determinant as to the immunomodulatory (anti-inflammatory) nature of MSCs in response to a biomaterial, assessing a wider variety of cytokines may further elucidate the immunomodulatory and therefore regenerative potential of MSCs when combined with RpCol. Furthermore, investigating the immune response to RpCol hydrogels, and the interplay of MSCs with the immune system within the environment of RpCol hydrogels, may provide a good indication of the nature of the host response that would be elicited *in vivo* and thus the potential of RpCol hydrogels to be utilised in a wide range of therapies (Salthouse et al., 2023). Specific regenerative medicine applications that this developed hydrogel system may be utilised in could range from soft tissue regeneration (such as neural regeneration), bone and cartilage engineering for the treatment of related diseases (such as osteoporosis and osteoarthritis), organ regeneration and transplantation (such as lung, kidney, liver, cardiac, and skin wound healing), to potentially being utilised in cellular therapies to treat immune-related and inflammatory diseases/disorders due to the immunomodulatory ability of MSCs for example (Han et al., 2019; Wang J. et al., 2020; Margiana et al., 2022; Petrosyan et al., 2022). However, the suitability of these RpCol hydrogels for any of these applications, or indeed for any other specific application, requires further extensive investigation to elucidate their full potential.

## 3 Conclusion

This study investigated the properties of various RpCol hydrogel formulations crosslinked with a range of chemical agents and assessed their potential to be utilised in regenerative medicine applications. RpCol alone did not form stable hydrogels. A variety of chemical crosslinking agents were therefore added to produce stable RpCol hydrogels to enable their application in regenerative medicine. 4-arm PEG-GAS formed unstable RpCol hydrogels that were fully degraded within 3 days, whereas hydrogels produced from 10 kDa 4- and 8-arm PEG-SCM had improved stability, with 8-arm PEG-SCM-crosslinked RpCol hydrogels demonstrating significantly less hydrolytic degradation. At all ratios tested, EDC/NHS and EDC/sNHS at an EDC concentration of 1% did not produce robust enough RpCol hydrogels, however, hydrogel stability was increased when using EDC concentrations of both 5% and 10%. SEM demonstrated a wide variety of pore diameter distributions of RpCol hydrogels formed with the different chemical crosslinking agents, however, the rate of glucose uptake of RpCol hydrogels indicated that the differences in pore sizes did not have a significant effect on the rate of diffusion. Rheological analyses indicated that RpCol hydrogels crosslinked with both 16% 8-arm PEG-SCM and EDC/sNHS 5% 1:1 were relatively strong, with crosslinking with 16% 8-arm PEG-SCM facilitating a quicker gelation time. The endotoxin levels of discontinued RpCol exceeded regulatory limits, however, the endotoxin levels of new RpCol were <0.5 EU/mL, thus supporting the potential use of RpCol *in vivo*. Encapsulating TERT-hMSCs within RpCol hydrogels crosslinked with EDC/sNHS 5% 1:1 induced MSC cell death and therefore we concluded that these hydrogels are unsuitable for future work. 16% 8-arm PEG-SCM-crosslinked RpCol hydrogels induced a significant increase in the metabolic activity of encapsulated MSCs and caused very minimal cell death. Interestingly, there was a significant increase of IL-10 production by TERT-hMSCs encapsulated within both EDC- and PEG-crosslinked RpCol hydrogels, therefore it is difficult to conclude the true effects of these results. Overall, 8-arm PEG-SCM-crosslinked RpCol hydrogels display immense potential to be utilised in a wide range of regenerative medicine applications. Depending on the application, different immunomodulatory molecules could be incorporated into RpCol hydrogels to modulate the host immune response and promote tissue regeneration.

## 4 Materials and methods

### 4.1 RpCol hydrogel preparation

**FIGURE 8:**
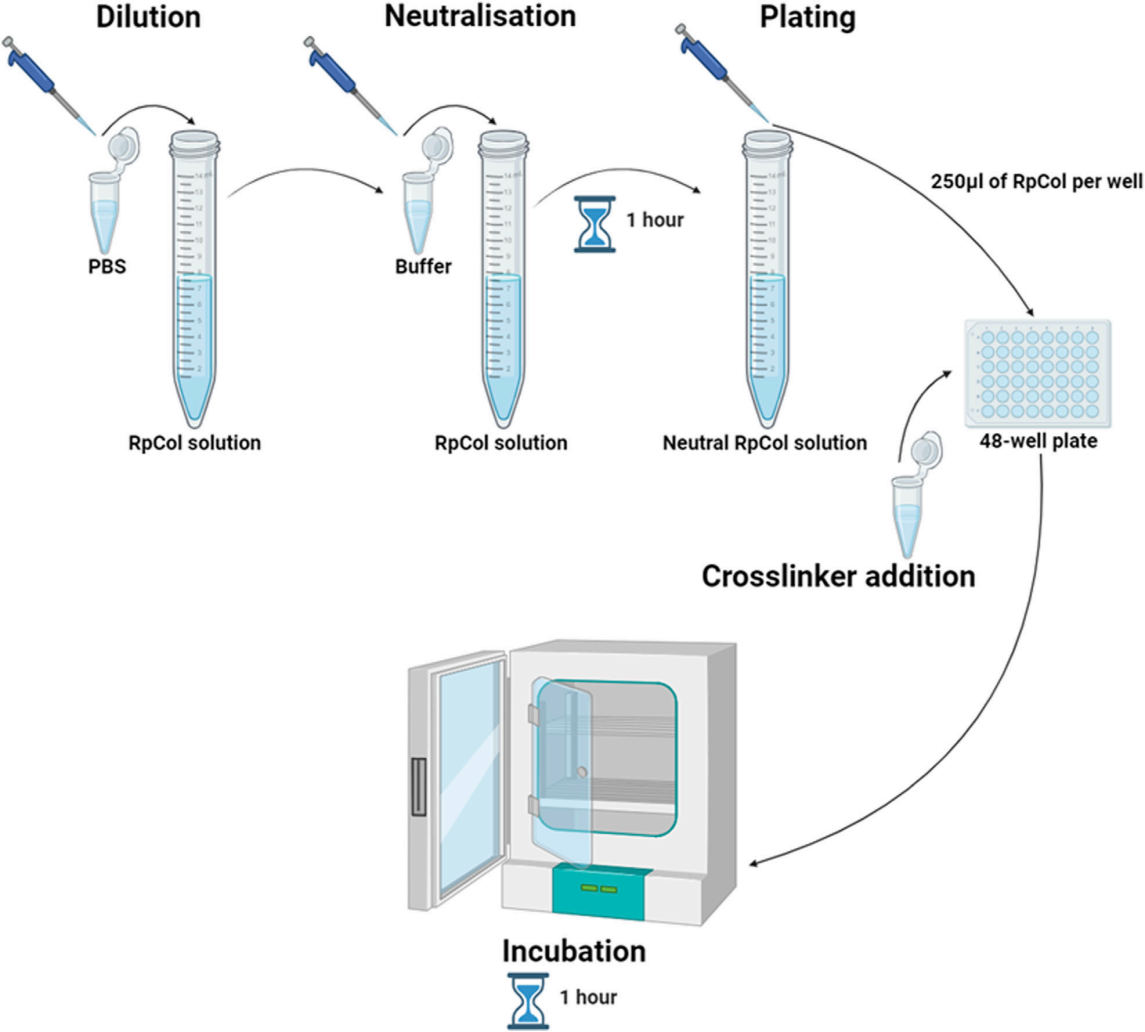
Schematic diagram illustrating the preparation of RpCol hydrogels; dilution of RpCol solution to 5 mg/mL with PBS, neutralisation of RpCol solution for 1 h following the addition of neutralising buffer (120 μL of neutralising buffer per 1 mL of RpCol solution, giving an RpCol concentration of 4.46 mg/mL), plating of 250 μL per well of neutralised RpCol solution into a 48-well plate, chemical crosslinking agent addition (in their respective volumes) to the neutralised collagen solution in the 48-well plate to form hydrogels, and incubation at 37°C for 1 h. Created in BioRender.com.

*Rhizostoma pulmo* collagen {RpCol [ECM collagen type 0 (Faruqui et al., 2023)]} was kindly provided by Jellagen Limited and stored in acidic solutions at either 6.74 mg/mL or 6.5 mg/mL. Specific neutralising buffers (compositions are proprietary information) and 20 kDa 4-arm PEG-GAS (polyethylene glycol succinimidyl glutaramide ester) were provided by Jellagen Limited in a hydrogel kit (discontinued). 48-well plates (Greiner Bio-One, 677180) were used for hydrogel preparations. A schematic diagram illustrating the preparation of RpCol hydrogels is demonstrated in Figure 8, while Table 3 details the exact volumes of chemical crosslinking agents required to prepare each specific RpCol hydrogel. Prior to use, RpCol solution was diluted with Dulbecco’s Phosphate Buffered Saline (PBS) (Sigma-Aldrich, D8537) to 5 mg/mL, followed by the addition of neutralising buffer (120 μL of neutralising buffer was added per 1 mL of RpCol solution, giving an RpCol concentration of 4.46 mg/mL) and RpCol was left to neutralise for 1 h. Methodology for the addition of PEG (polyethylene glycol)- and EDC(*N*-(3-Dimethylaminopropyl)-N’-ethylcarbodiimide hydrochloride-based chemical crosslinking agents was adapted from Fernandes-Cunha et al (2020). Chemical crosslinking agents were solubilised in PBS to 100 mg/mL (w/v); and for 4%, 8%, and 16% (v/v) PEG-SCM (polyethylene glycol succinimidyl carboxyl methyl ester) hydrogels (CreativePEGWorks, 4-arm (PSB-486), 8-arm (PSB-841)), 10, 20, and 40 µL of PEG-SCM, respectively, was added to 250 µL of RpCol. The same practice was applied to EDC (Sigma-Aldrich, E7750), NHS (*N*-Hydroxysuccinimide) (Sigma-Aldrich, 130672), and sNHS (*N*-Hydroxysulfosuccinimide sodium salt) (Sigma-Aldrich, 56485), whereby an EDC/NHS 1% (v/v) 2:1 (by volume) hydrogel consisted of 250 µL of RpCol, 2.5 µL of EDC, and 1.25 µL of NHS. Methodology for the addition of genipin (Sigma-Aldrich, G4796) was adapted from Riacci et al. (2021). Genipin was solubilised in PBS to 10 mg/mL (w/v); and for 1, 2.5, and 5 mM genipin hydrogels, 5.7, 14.1, and 28.3 μL, respectively, was added to 250 μL of RpCol. RpCol was first pipetted into 48-well plates, followed by the addition of a chemical crosslinking agent. All hydrogels were briefly mixed using a pipette tip and incubated at 37°C for 1 h. Photos of final RpCol hydrogels used in cell studies is shown in Supplementary Figure S1.

**TABLE 3:** Volume of chemical crosslinking agents added to 250 µL of RpCol solution to form RpCol hydrogels.

Sample	Volume of crosslinker added to 250 µL of RpCol (µL)
Uncrosslinked RpCol	0
4-arm PEG-SCM 4%	10
4-arm PEG-SCM 8%	20
4-arm PEG-SCM 16%	40
8-arm PEG-SCM 4%	10
8-arm PEG-SCM 8%	20
8-arm PEG-SCM 16%	40
EDC/NHS 1% 1:1	2.5
EDC/NHS 5% 1:1	12.5
EDC/NHS 10% 1:1	25
EDC/sNHS 1% 1:1	2.5
EDC/sNHS 5% 1:1	12.5
EDC/sNHS 10% 1:1	25
Genipin 1 mM	5.7
Genipin 2.5 mM	14.1
Genipin 5 mM	28.3

### 4.2 Freeze-drying

Chemical crosslinking agents were added to 500 µL of RpCol (in their equivalent concentrations and ratios as when adding to 250 μL of RpCol) to form RpCol hydrogels and incubated at 37°C for 1 h. The 48-well plate lid was replaced with parafilm, five small holes per well were made with a needle, and the plate was transferred to a freezer at −20°C. After 3 days, the plate was transferred to a Christ ALPHA 1-2 LDplus freeze-dryer starting at −25°C and reaching −55°C, with the process lasting 48 h.

### 4.3 Fourier-transform infrared spectroscopy (FTIR)

An FTIR transmittance spectra at 4,000–1,000 cm^−1^ was measured using an Agilent Technologies Cary 630 FTIR spectrometer equipped with diamond attenuated total reflection (ATR). Freeze-dried samples were used to minimise the interference of water molecules. A background scan was taken before every sample.

### 4.4 Hydrolytic degradation

Following chemical crosslinking agent addition, RpCol was incubated at 37°C for 1 h. RpCol hydrogels were removed from the 48-well plate, the initial mass (day 0) was measured, and RpCol hydrogels were returned to the plate and incubated at 37°C. Measurements of mass were taken on day 1, 2, 3, and 7. 250 µL of PBS was added to hydrogels after day 0 and 3 measurements to prevent hydrogel dehydration and mimic physiological conditions.

### 4.5 Scanning electron microscopy (SEM) imaging

Freeze-dried samples were loaded into a Hitachi TM3030 SEM and sliced horizontally to image the cross-sections. Images were taken at × 100 magnification. ImageJ software was used to determine pore diameters, with 60 measurements taken for each condition.

### 4.6 Glucose diffusion assay

Following hydrogel formation, hydrogels were incubated at 37°C for 1 h. Fluorescent d-glucose analogue 2-NBDG (2-(*N*-(7-Nitrobenz-2-oxa-1,3-diazol-4-yl)Amino)-2-Deoxyglucose) (Invitrogen^TM^, N13195) was diluted to 0.06845 mg/mL as specified by da Conceicao Ribeiro et al. (2018), and 250 µL was added to each hydrogel. Hydrogels were in contact with 2-NBDG solution for 0, 5, 15, 30, 60, or 120 min, whereupon the hydrogels were removed and transferred to a fresh 48-well plate. 400µL of PBS was added to each hydrogel and the plate was covered in aluminium foil and stored at 4°C for 24 h. The hydrogels were removed from the plate, 200 µL of the remaining solution was transferred to a 96-well plate (Greiner Bio-One, 655090) and absorbance was read on a FLUOstar Omega (BMG Labtech) microplate reader at 485–590 nm immediately after assay completion. A standard curve was generated on GraphPad Prism 9 software to determine the concentration of 2-NBDG absorbed. 120 min was assumed as 100% glucose uptake.

### 4.7 Rheological analyses

Rheological measurements were performed on a modular compact rheometer (MCR302e, Anton Paar) at 37°C in a plate-plate geometry, with a diameter of 20 mm and a gap between the two plates of 1 mm. 500 μL of RpCol was pipetted directly onto the rheometer plate, followed by the addition of a chemical crosslinking agent (in the equivalent concentration/ratio as when adding to 250 μL of RpCol) and a brief mix using a pipette tip. For amplitude sweeps, the storage modulus (G’) and loss modulus (G”) were measured in oscillation mode from 1% to 1,000% shear strain at a frequency of 1 Hz. For time sweeps, the storage modulus (G’) and loss modulus (G”) were measured over 30 min at a frequency of 1 Hz.

### 4.8 Endotoxin quantification

Endotoxin content of RpCol was determined using the Pierce™ Chromogenic Endotoxin Quant Kit (Thermo Fisher Scientific, A39552) following the manufacturer’s protocol. Optical density at 405 nm was measured using a FLUOstar Omega (BMG Labtech) microplate reader immediately after assay completion. A standard curve was generated on GraphPad Prism 9 software to determine the concentration of endotoxin.

### 4.9 Cell culture of immortalised human mesenchymal stem/stromal cells (TERT-hMSCs)

TERT-hMSCs, kindly provided by The University of York, were cultured in filtered T75 or T175 flasks with Dulbecco’s Modified Eagle’s Medium (DMEM) (Gibco, 11960-044) supplemented with 10% foetal bovine serum (FBS) (Gibco, 10500-064), 1% GlutaMax™ (Fisher Scientific, 11574466), and 1% Penicillin-Streptomycin (Sigma-Aldrich, P0781), filtered through a 0.22 µm filter. Cells were washed with PBS before the addition of 0.25% trypsin-EDTA (Sigma-Aldrich, T4049). Cell suspensions were centrifuged in a falcon tube at 1200 rpm for 5 min. Cells were incubated at 37°C with 5% CO_2_. TERT-hMSCs were used from passage 77-90. All cell work was performed in a Class II Biosafety Cabinet.

### 4.10 TERT-hMSC encapsulation within RpCol hydrogels

Cells were encapsulated at a density of 5 × 10^5^ cells/mL, the positive control was 5,000 cells on tissue culture plastic, and two negative controls were hydrogels with DMEM and DMEM alone. Cells were encapsulated by resuspending at 4 × 10^6^ cells/mL and adding 1–7 mL of RpCol (to a final RpCol concentration of 4.46 mg/mL). Hydrogels and controls were incubated at 37°C and 500 µL of DMEM was added after 15 min. DMEM was replaced at 60- and 100-min post-encapsulation and was changed daily.

### 4.11 Cellular morphology and viability imaging

Morphology was analysed using phalloidin (Invitrogen^TM^, R37112) and DAPI (Invitrogen^TM^, P36971) staining. Wells were washed with 500 µL of PBS and 500 µL of 4% paraformaldehyde (PFA) (Thermo Fisher Scientific, J19943-K2) was added for 30 min at room temperature. PFA was removed using three, 5-min washes with 500 µL of PBS at 37°C. Phalloidin stock was made by diluting two drops per ml in PBS, of which 250 µL was added to each well for a 20-min incubation at 37°C. After a PBS wash, 100 µL of DAPI was added to each well for 20 min at room temperature.

Live and dead cells were imaged using a Blue/Green Cell Viability Imaging Kit (Invitrogen^TM^, R37609), respectively. Reagents were diluted in PBS, two drops each per ml. Wells were washed with 500 µL of PBS, and 250 µL of the Live/Dead stock solution was added to each well and incubated at 37°C for 15 min. Cells were imaged on an Evos microscope at × 20 magnification.

### 4.12 Assessment of cellular metabolic activity

PrestoBlue^TM^ measures the extent of the metabolism of resazurin into resorufin. Wells were washed with 500 µL of PBS, PrestoBlue^TM^ (Invitrogen^TM^, A13261) was diluted in DMEM in a 1:9 ratio, and 500 µL was added to each condition and incubated for 2 h at 37°C. 200 µL from each well was transferred to a 96-well plate (Greiner Bio-One, 655090) and fluorescence was read on a FLUOstar Omega (BMG Labtech) at 485–590 nm immediately after assay completion. A standard curve was generated on GraphPad Prism 9 software to determine the relative extent of metabolism. Metabolic assays were always protected from light.

### 4.13 Cytokine analysis

Interleukin (IL)-10 levels of cell-free supernatants from cellular experiments were analysed with an IL-10 Human ELISA (enzyme-linked immunosorbent assay) kit (Invitrogen^TM^, BMS215-2) following the manufacturer’s protocols. A standard curve was generated on GraphPad Prism 9 software to determine the concentration of IL-10.

### 4.14 Statistical analyses

Graphs were plotted and statistical analyses were performed using GraphPad Prism 9 software. Asterix represent degree of significance; *p* < 0.05*, *p* < 0.01**, *p* < 0.001***, *p* < 0.0001****. 2-way ANOVA tests were used to analyse between conditions. Paired t-tests were used to analyse the same chemical crosslinking agent between different days. Unpaired t-tests were used to analyse different chemical crosslinking agents between the same day. Data presented as mean ± standard error of the mean of two independent experiments performed in triplicate or three independent experiments performed in triplicate (specified in each figure legend where statistical analyses were performed).

## Data Availability

The datasets presented in this study can be found in online repositories. The names of the repository/repositories and accession number(s) can be found below: https://data.ncl.ac.uk.
