# Supplementary material for: Amine-reactive crosslinking enhances type 0 collagen hydrogel properties for regenerative medicine
Source: Front Bioeng Biotechnol. 2024 Jul 26;12:1391728. doi: 10.3389/fbioe.2024.1391728 (PMC11310005; doi:10.3389/fbioe.2024.1391728)
Supplement: Supplementary file 1 [file DataSheet1.docx]

Supplementary Material

**Table S1.** Values (to 3 significant figures) generated from amplitude sweeps of RpCol hydrogels crosslinked with; 4%, 8%, and 16% 4- and 8-arm PEG-SCM, 1%, 5%, and 10% EDC/NHS and EDC/sNHS 1:1, and 1mM, 2.5mM, and 5mM genipin.

| **Sample** | **LVR** | **Yield point** | | **Crossover point** |
| --- | --- | --- | --- | --- |
|  | **Shear strain (%)** | **Shear strain (%)** | **G’, G”** | **Shear strain** |
|  |  |  | **(Pa)** | **(%)** |
| 4-arm PEG-SCM 4% | 1.00-30.5 | 30.5 | 19.3, 1.40 | ~300 |
| 4-arm PEG-SCM 8% | 1.00-30.5 | 30.5 | 15.8, 1.31 | ~400 |
| 4-arm PEG-SCM 16% | 1.00-30.5 | 30.5 | 35.9, 1.78 | ~400 |
| 8-arm PEG-SCM 4% | 1.00-42.0 | 42 | 1.80, 0.182 | ~600 |
| 8-arm PEG-SCM 8% | 1.00-30.5 | 30.5 | 39.4, 2.69 | ~600 |
| 8-arm PEG-SCM 16% | 1.00-42.0 | 42 | 51.2, 2.80 | ~400 |
| EDC/NHS 1% 1:1 | 1.00-2.41 | 2.41 | 8,45, 1.04 | ~100 |
| EDC/NHS 5% 1:1 | 1.00-30.5 | 30.5 | 18,4, 1.74 | ~400 |
| EDC/NHS 10% 1:1 | 1.75-42.0 | 42 | 2.27, 0.296 | ~300 |
| EDC/sNHS 1% 1:1 | 1.00-16.2 | 16.2 | 34.0, 3.94 | ~300 |
| EDC/sNHS 5% 1:1 | 1.00-42.0 | 42 | 26.6, 2.87 | ~550 |
| EDC/sNHS 10% 1:1 | 1.28-109 | 109 | 0.247, 0.0772 | ~400 |
| Genipin 1mM | 1.00-30.5 | 30.5 | 6.34, 0.789 | ~400 |
| Genipin 2.5mM | 1.00-109 | 109 | 0.442, 0.0899 | ~400 |
| Genipin 5mM | 2.41-79.2 | 79.2 | 0.160, 0.030 | ~400 |

**Table S2.** Values (to 3 significant figures) generated from time sweeps of RpCol hydrogels crosslinked with; 4%, 8%, and 16% 4- and 8-arm PEG-SCM, 1%, 5%, and 10% EDC/NHS and EDC/sNHS 1:1, and 1mM, 2.5mM, and 5mM genipin.

| Sample | Start value | | End value | |
| --- | --- | --- | --- | --- |
|  | **G’**  **(Pa)** | **G”**  **(Pa)** | **G’**  **(Pa)** | **G”**  **(Pa)** |
| 4-arm PEG-SCM 4% | 8.89 | 0.917 | 113 | 9.55 |
| 4-arm PEG-SCM 8% | 63.3 | 3.89 | 552 | 39.9 |
| 4-arm PEG-SCM 16% | 24.8 | 1.35 | 683 | 67.1 |
| 8-arm PEG-SCM 4% | 16.7 | 2.39 | 550 | 35.0 |
| 8-arm PEG-SCM 8% | 19.5 | 0.385 | 489 | 33.0 |
| 8-arm PEG-SCM 16% | 18.6 | 1.44 | 123 | 5.56 |
| EDC/NHS 1% 1:1 | 4.68 | 1.76 | 68.3 | 4.91 |
| EDC/NHS 5% 1:1 | 2.68 | 0.778 | 113 | 8.23 |
| EDC/NHS 10% 1:1 | 2.49 | 0.651 | 143 | 6.49 |
| EDC/sNHS 1% 1:1 | 4.04 | 0.619 | 159 | 13.3 |
| EDC/sNHS 5% 1:1 | 4.37 | 0.822 | 121 | 4.61 |
| EDC/sNHS 10% 1:1 | 1.84 | 0.235 | 157 | 5.83 |
| Genipin 1mM | 3.01 | 0.712 | 299 | 23.2 |
| Genipin 2.5mM | 1.20 | 0.274 | 54.1 | 1.72 |
| Genipin 5mM | 3.16 | 1.39 | 139 | 12.8 |


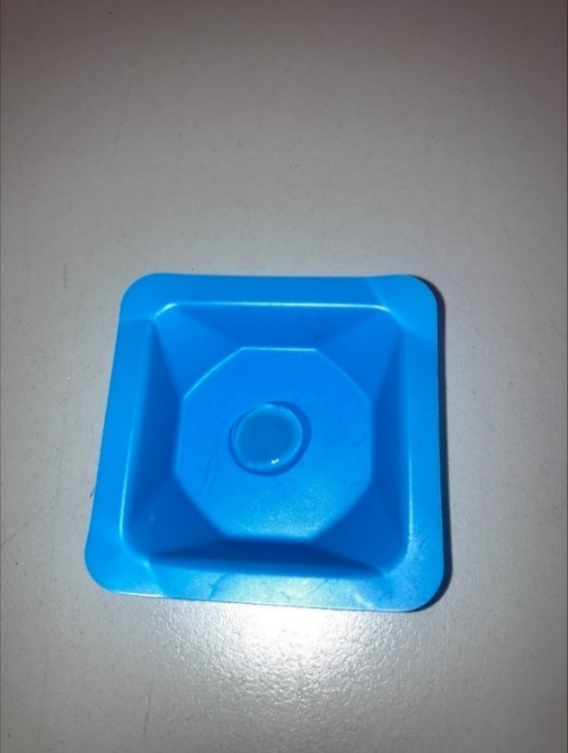


**A)**

**B)**


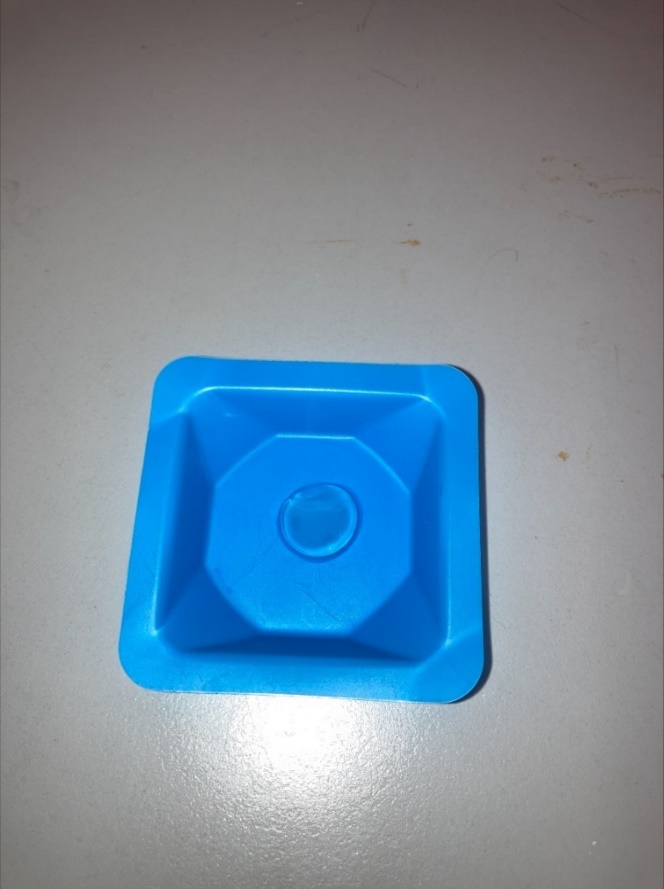


**Figure S1.** Photos of final RpCol hydrogels used in cell studies. **A)** 16% 8-arm PEG-SCM-crosslinked RpCol hydrogel. **B)** EDC/sNHS 5% 1:1-crosslinked RpCol hydrogel.
